# Phenotypic and molecular characterization of IMP-producing Enterobacterales in Spain: Predominance of IMP-8 in *Klebsiella pneumoniae* and IMP-22 in *Enterobacter roggenkampii*

**DOI:** 10.3389/fmicb.2022.1000787

**Published:** 2022-09-28

**Authors:** Javier E. Cañada-García, Natalin Grippo, Eva Ramírez de Arellano, Verónica Bautista, Noelia Lara, Ana María Navarro, Teresa Cabezas, Nora Mariela Martínez-Ramírez, Silvia García-Cobos, Jorge Calvo, Emilia Cercenado, Belén Aracil, María Pérez-Vázquez, Jesús Oteo-Iglesias, the Spanish IMP Study Group

**Affiliations:** ^1^Laboratorio de Referencia e Investigación en Resistencia a Antibióticos e Infecciones Relacionadas con la Asistencia Sanitaria, Centro Nacional de Microbiología, Instituto de Salud Carlos III, Madrid, Spain; ^2^Centro de Educación Médica e Investigaciones Clínicas “Norberto Quirno”, Buenos Aires, Argentina; ^3^Servicio de Microbiología, Hospital de Poniente, Almería, Spain; ^4^Servicio de Microbiología, Hospital Universitario de Guadalajara, Guadalajara, Spain; ^5^CIBER de Enfermedades Infecciosas (CIBERINFEC), Spanish Network for Research in Infectious Diseases (REIPI), Instituto de Salud Carlos III, Madrid, Spain; ^6^Servicio de Microbiología, Hospital Universitario Marqués de Valdecilla, Santander, Spain; ^7^Servicio de Microbiología, Hospital Universitario Gregorio Marañón, Madrid, Spain; ^8^CIBER de Enfermedades Respiratorias (CIBERES), Instituto de Salud Carlos III, Madrid, Spain

**Keywords:** antimicrobial resistant bacteria, carbapenem resistance, whole genome sequencing, surveillance, IMP carbapenemase, Enterobacterales

## Abstract

**Objectives:**

Little is known about IMP-producing Enterobacterales (IMP-Ent) in Europe. We analyzed at genomic and phenotypic level IMP-Ent isolates circulating in Spain in a 9-year period.

**Materials and methods:**

IMP-Ent isolates submitted to our reference laboratory were included. Antibiotic susceptibility was performed using microdilution method (EUCAST), and IMP-carbapenemase activity was measured with carbapenemase inhibitors, the β-CARBA method, the modified Hodge test (MHT), and the modified carbapenemase inhibition method (mCIM). All isolates collected were sequenced for high-resolution single-nucleotide polymorphism (SNP) typing, core genome multilocus sequence typing (cgMLST), and resistome analysis.

**Results:**

Fifty IMP-Ent isolates, collected from 19 hospitals in 13 Spanish provinces, were detected: *Klebsiella pneumoniae* (IMP-Kpn) (24; 48%), *Enterobacter roggenkampii* (13; 26%), *Enterobacter hormaechei* (8, 16%), *Klebsiella oxytoca* (two; 4%), *Enterobacter asburiae* (one, 2%), *Serratia marcescens* (one; 2%) and *Escherichia coli* (one; 2%). All isolates were positive by the MHT and β-CARBA tests; 48 (96%) were mCIM positive; 12 (24%) and 26 (52%) displayed positive inhibition with dipicolinic (meropenem) and EDTA (ertapenem), respectively. Five IMP-carbapenemase types were identified: IMP-8 (22; 44%), IMP-22 (17; 34%), IMP-13 (7; 14%), IMP-28 (two; 4%), and IMP-15 (two; 4%), predominating IMP-8 in *K. pneumoniae* and IMP-22 in *E. roggenkampii*. IMP-28 was exclusively identified in *K. oxytoca* and IMP-15 in *E. hormaechei*. Predominant STs were ST405 (29.2%), ST15 (25%) and ST464 (20.8%) in IMP-Kpn; ST96 (100%) in *E. roggenkampii* and ST182 (62.5%) in *E. hormachei*. Colistin and amikacin were the most active non-carbapenem antibiotics against IMP-Ent.

**Conclusion:**

IMP-Ent isolates remain infrequent in Spain, although in recent years have been circulating causing nosocomial outbreaks, being IMP-8-producing *K. pneumoniae* and IMP-22-producing *E. roggenkampii* the most frequently detected in this study. Inhibition with EDTA or dipicolinic acid presented false negative results in some IMP-producing strains. Active microbiological and molecular surveillance is essential for a better comprehension and control of IMP-Ent dissemination.

## Introduction

Carbapenemase-producing Enterobacterales (CPE) have emerged as a major global health threat, mainly due to spread of OXA-48, KPC and NDM carbapenemases (Tzouvelekis et al., 2012). In contrast, imipenemase (IMP)-type carbapenemases, although common in *Pseudomonas aeruginosa*, are infrequently detected and thus poorly understood in Enterobacterales species (Zhao and Hu, 2011). Worldwide, Asia and Oceania display the highest prevalence of IMP-producing carbapenem-resistant Enterobacterales (IMP-Ent; Zhao and Hu, 2011; Matsumura et al., 2017; Han et al., 2020); which are scarce in Europe. There is a lack of information on the molecular characterization by whole genome sequencing (WGS) of a large collection of IMP-producing Enterobacterales. The European Survey on Carbapenemase-Producing *Enterobacteriaceae* (EuSCAPE) reported only three IMP-producing *Klebsiella pneumoniae* isolates out of 684 (0.4%) carbapenemase-encoding organisms (David et al., 2019).

Moreover, many rapid commercially available tests for carbapenemase identification (e.g., immunochromatography, PCR) do not detect *bla*_IMP_ genes, or include only specific types (e.g., *bla*_IMP-1_) that are not necessarily the most prevalent. Further, due to their low frequency, a detailed understanding of IMP-Ent behaviour in phenotypic tests for carbapenemase characterization, such as the modified Hodge test (MHT) test, colorimetric assays, the carbapenemase inhibition method, and use of specific inhibitors, is lacking. Thus, there is substantial risk for under-detection of IMP-Ent.

The aim of this multicenter nation-wide study was to provide knowledge about the IMP-Ent isolates, characterizing their molecular epidemiology and resistance mechanisms (including WGS analysis) and evaluating different phenotypic methods for carbapenemases detection in these bacteria in Spain.

## Materials and methods

### Study design and isolates

This study was performed by the unrestricted and non-mandatory national Spanish Antibiotic Resistance Surveillance Programme, operated by our official public health Institute (Instituto de Salud Carlos III). We included IMP-Ent isolates submitted to our reference lab from January 2012 to December 2021, analyzing only the first isolate obtained from each patient. Initial assays were performed at each participating hospital using standard microbiological methods. CPE isolates were identified according to the European Committee on Antimicrobial Susceptibility Testing (EUCAST)-established meropenem cut-off values for CPE (European Committee on Antimicrobial Susceptibility Testing [EUCAST], 2017) and phenotypic confirmation of carbapenemase production was verified using at least one EUCAST-recommended method (European Committee on Antimicrobial Susceptibility Testing [EUCAST], 2017).

### Drug susceptibility testing and phenotypic methods for carbapenemases detection

Antibiotic susceptibility testing was performed by micro-dilution (DKMGN panel, Thermo Fisher Scientific, Waltham, MA, United States) and interpreted according to EUCAST breakpoints (European Committee on Antimicrobial Susceptibility Testing [EUCAST], 2017, 2022). Carbapenemase activity of IMP-Ent was tested using meropenem plus dipicolinic acid, phenyl-boronic acid, and cloxacillin inhibitors (European Committee on Antimicrobial Susceptibility Testing [EUCAST], 2017) (Rosco Diagnostica A/S, Taastrup, Denmark), and ertapenem plus 10 μl of EDTA inhibitor; the β-CARBA colorimetric detection method (Bio-Rad, Marnes-la-Coquette, France); the MHT, with meropenem discs containing 600 mg cloxacillin; and the modified carbapenemase inhibition method (mCIM) (Pierce et al., 2017).

### Genomic library preparation and sequence analysis

Genomic library preparation and sequence analysis were conducted as described (Pérez-Vázquez et al., 2019). Raw sequence data were submitted to the European Nucleotide Archive (PRJEB54568). Quality of short reads was assessed using FASTQC, and assembly into contigs was performed with Unicycler 0.4.8 (Wick et al., 2017). Quality of the assembly was assessed with QUAST^1^. Prokka v1.14-beta (Seemann, 2014) was used for automatic *de novo* assembly annotation. Mash v2.0 (Ondov et al., 2016) was used to analyze the similarity of each sequence to the genomes in RefSeq bacterial database;^2^ finally, we report the species and subspecies if assigned as the top matched with the smallest distance. For all top matched sequences with Mash, ANI distances against reference genomes of the main subspecies were calculated with OthoANIu toll (Yoon et al., 2017); the identification was considered the one of the reference in the pair with highest ANI value applying the threshold described by Sutton et al. (2018). Mash distances and ANI results are reported in Supplementary Table S1. All the *Klebsiella* species and subspecies were confirmed with Kleborate.^3^

### Phylogenetic analysis

Prokka v.1.14 (Seemann, 2014) was used to annotate *de novo* assemblies, these annotated assemblies were used as input for Roary v3.13.0 (Page et al., 2015). For *Klebsiella pneumoniae* and *Enterobacter cloacae complex* alignments of 3.589 and 686 core genes (present in ≥ 99% of isolates) comprising 3.533.275 bp and 672.747 bp were generated, respectively. Variable positions of the alignment were extracted with Snp sites (Page et al., 2016) in both species (64.568 and 67.619 SNPs, respectively), finally pairwise distances were calculated with MegaX v10.0.5 (Kumar et al., 2018).

Sequence types (STs) were calculated according to multilocus sequence typing (MLST) schemes of the Institut Pasteur for *K. pneumoniae*^4^ and *E. cloacae* complex using Ariba v2.6.2 (Hunt et al., 2017). Core-genome MLST (cgMLST) consisting of 2.538 targets for *K. pneumoniae* a provided by SeqSphere+3.5.0 (Ridom, Germany), was performed. For global *E. cloacae* complex, *Enterobacter hormaechei* and *Enterobacter roggenkampii* trees *ad hoc* schemes were created using the MLST+ target definer with the default parameters and a reference sequence: *Enterobacter cloacae subsp. cloacae* ATCC 13047 (accession no NC_014121), *E. hormaechei* (accession no NC_021046) and *E. roggenkampii* (accession no NZ_CP022148.1). A total of 38, 24 and 26 NCBI RefSeq genomes for global *E. cloacae* complex, *E. hormaechei* and *E. roggenkampii*, respectively, were used as query genomes to validate in a pairwise comparison using BLAST. Genes selected in the cgMLST were common in all query genomes and they presented a percentage of identity higher that 90%. The final cgMLST schemes consisted in 631 targets genes and 4.322 accessory genes for *E. cloacae* complex, 2.123 and 1.798 for *E. hormaechei* and 2.466 and 1.951 for *E. roggenkampii*. The average percentage of good targets included in the cgMLST was 99.3, 98.9 and 99.7% for *E. cloacae* complex, *E. hormaechei* and *E. roggenkampii*, respectively.

#### Analysis of antimicrobial resistance, virulence genes, and capsular locus

Antibiotic resistance genes were analyzed by SRST2 (Inouye et al., 2014) using the ARG-ANNOT database (Gupta et al., 2014) and ResFinder (CGE server)^5^, with ID thresholds of 100% for β-lactamase variants (except for chromosomal AmpC genes), and 98% for the other resistance genes. The K-locus and virulence genes were characterized in IMP-producing *K. pneumoniae* isolates using Kleborate.^6^ The presence of *ybt*, *clb,* and *iuc* was used to assign a virulence score, as described (Lam et al., 2021).

## Results and discussion

### Bacterial isolates, patients and carbapenemase types

During the study period, 50 non-duplicated IMP-Ent isolates from 19 Spanish hospitals in 13 provinces were submitted to the reference laboratory (Table 1), which corresponded to 0.4% of the 12,038 carbapenemase-producing Enterobacterales isolates detected in this laboratory. Most of IMP-Ent isolates were from men (64%) and people over 65 years old (52%). No temporal significant trend was detected in the proportion of IMP-Ent among total CPE identified by our laboratory by year, which ranged between 2.1% in 2012 and 0.2% in 2015.

**Table 1:** Main microbiological and epidemiological features of the 50 IMP-producing Enterobacterales isolates included in this study.

Species (*n*)	CBPs (*n*)	MLST	ESBL/qAmpC (*n*)	Year (*n*)	Province	Sample
*K. pneumoniae* (24)	IMP-8 (14)	ST405 (7)	*bla*_CTX-M-15_ (6)	2014 (6), 2016 (1)	Almería	Rectal (6), respiratory tract (1)
		ST464 (5)	*bla*_CTX-M-15_ (2)	2012 (3), 2013 (2)	Almería	Rectal (3), urine (2)
		ST15 (2)	*bla*_CTX-M-15_ (2)	2015	Málaga	Urine (1), respiratory tract (1)
	IMP-22 (6)	ST15 (4)	*bla*_SHV-134_ (1)	2013 (2), 2014 (1), 2015 (1)	Madrid	Urine (3), blood (1)
		ST3157 (1)	N	2018	Pontevedra	Wound exudate
		ST788 (1)	N	2017	Segovia	Urine
	IMP-8 + OXA-48 (2)	ST378	*bla*_FOX-5_ (1)	2017	Alicante	Wound exudate
	IMP-22 + KPC-3 (2)	ST11	*bla*_GES-7_ (1)	2020	Madrid	Blood (1), wound exudate (1)
*E. roggenkampii* (13)	IMP-22 (7)	ST96	N	2012 (2), 2013 (3), 2017 (1)	Guadalajara (5), Ciudad Real (1)	Urine (3), blood (1), abscess (1), rectal (1)
	IMP-8 (4)	ST96	N	2017 (1), 2018 (3)	Navarra (3), Toledo (1)	Rectal (3), respiratory tract (1)
	IMP-22 + VIM-1 (2)	ST96	N	2018	Guadalajara	Rectal (2)
*E. hormaechei* (8)	IMP-13 (5)	ST182 (4)	*bla*_CTX-M-15_ (1)	2015 (1), 2016 (1), 2017 (1), 2021 (1)	Madrid (3), Cantabria (1)	Rectal (1), urine (2), ascitic fluid (1)
		ST90 (1)	*bla*_CTX-M-9_	2013	Vizcaya	Blood
	IMP-15 (2)	ST68 (1)	N	2014	Las Palmas	BAL
		ST182 (1)	N	2015	Madrid	Rectal
	IMP-8 (1)	ST68	N	2018	Toledo	Rectal
*E. asburiae* (1)	IMP-13 (1)	ST484	N	2014	Vizcaya	BAL
*K. oxytoca* (2)	IMP-28 (2)	New	N	2016	Madrid	Urine
*S. marcescens* (1)	IMP-8	ND	N	2018	Valladolid	Urine
*E. coli* (1)	IMP-13	ST399	N	2021	Vizcaya	Rectal

CBPs, Carbapenemases; ESBL, Extended-spectrum β-lactamase; qAmpC, acquired AmpC β-lactamases; N, negative; ND, not determined; BAS, Bronchoaspirate; BAL, Bronchoalveolar lavage.

Of the 50 isolates, 32 (64%) produced clinical infections, including 16 (32%) urinary tract infections, 5 (10%) respiratory tract infections, 4 (8%) bacteraemia cases, and 7 (14%) other infections; the remaining 18 (36%) were from rectal samples.

The IMP-producing isolates included 24 (48%) strains of *K. pneumoniae* “*sensu stricto*”; 22 (44%) *E. cloacae* complex belonging to *E. roggenkampii* (13; 26%), *E. hormaechei* (8, 16%, five belonged to *E. hormaechei subsp xiangfangensis*, two to *E. hormaechei subsp. steigerwaltii* and one to *E. hormaechei subsp. oharae*), and *Enterobacter asburiae* (one, 2%) species; two (4%) *Klebsiella oxytoca*, one (2%) *Serratia marcescens,* and one (2%) *Escherichia coli* (Table 1; Supplementary Table S1). Outside of Asia and Oceania, little is known about IMP-Ent, and most isolates are from single cases or small outbreaks (Zhao and Hu, 2011; Matsumura et al., 2017; García-Castillo et al., 2018). A recently published collection of 935 carbapenem-resistant Enterobacterales from China contained 16 IMP-producing isolates (Han et al., 2020). Thus, our collection of IMP-Ent from Spain can provide valuable insights into the biology of European isolates. In addition, there are very few descriptions in the literature of carbapenemase-producing *E. roggenkampii* (Mateos et al., 2021; Umeda et al., 2021), and only one associated with IMP carbapenemases (Umeda et al., 2021).

Characterization of β-lactamases genes by DNA sequencing showed five *bla*_IMP_ genes encoding IMP-8 (22, 44%), IMP-22 (17, 34%), IMP-13 (7, 14%), IMP-28 (2, 4%), and IMP-15 (2, 4%) (Table 1). IMP-8 predominated in *K. pneumoniae* (16/22; 72.7%), whereas IMP-22 was most prevalent in *E. roggenkampii* (9/13; 69.2%) isolates. IMP-28 was only found in *K. oxytoca* (Table 1). An additional carbapenemase gene was observed in six isolates: two *K. pneumoniae* with IMP-8 plus OXA-48 of ST378, two *K. pneumoniae* with IMP-22 plus KPC-3 of ST11, and two *E. roggenkampii* with IMP-22 plus VIM-1 (Table 1).

IMP-8 is the type most frequently described in Enterobacterales worldwide, mainly in Asian countries such as Taiwan, where several outbreaks have been reported (Wang et al., 2015; Matsumura et al., 2017). However, nosocomial outbreaks of IMP-8-producing Enterobacterales have also occurred in other geographical regions, including those mediated by *K. oxytoca* in Spain (Vergara-López et al., 2013) and *E. coli* in Argentina (Elena et al., 2018); a specific search in PubMed did not provide any previous description of IMP-8 in *S. marcescens*. Conversely, IMP-22 has rarely been described, occurring mainly in Spain in *K. pneumoniae* and *E. coli* isolates (Miró et al., 2013; Ortega et al., 2016). Interhospital and community dissemination of IMP-22-producing *K. pneumoniae* has been described recently in the North of Portugal (Gonçalves et al., 2021). IMP-28 was first described in a *K. oxytoca* isolate in 2009 (Pérez-Llarena et al., 2012), from the same hospital in Madrid where the IMP-28-producing isolates of this study were collected 7 years later (2016). To the best of our knowledge, no previous cases of IMP-13 in *E. coli* have been reported; being an enzyme predominantly described in *P. aeruginosa* (Santella et al., 2010).

#### Antibiotic susceptibility testing and evaluation of different phenotypic methods for carbapenemase characterization in IMP-producing Enterobacterales

Frequencies of carbapenem susceptibility in *K. pneumoniae* and *E. cloacae* complex isolates were 66.6% (16/24) and 22.7% (5/22) for imipenem, respectively, and 62.5% (15/24) and 31.8% (7/22) for meropenem, respectively, with all susceptible isolates showing minimum inhibitory concentrations (MICs) of 1–2 mg/l (Table 2). Meropenem and imipenem MICs in non-susceptible isolates ranged 4–16 mg/l, being 4 mg/l the most frequent MIC (Table 3).

**Table 2:** Phenotypic features for detection of IMP production in IMP-producing Enterobacterales isolates.

Species (*n*)	CBPs (*n*)	Meropenem % S^a^	MIC_50_	MIC_90_	β-CARBA positive (%)	MHT positive (%)	mCIM positive (%)	DAI meropenem (%)	EDTA ertapenem (%)
Kpn (24)	IMP-8 (14)	12 (85.7)	2	4	14 (100)	14 (100)	14 (100)	1 (7.1)	13 (92.9)
	IMP-22 (6)	3 (50)	2	8	6 (100)	6 (100)	6 (100)	2 (33.3)	6 (100)
	IMP-8 + OXA-48 (2)	0	4	4	2 (100)	2 (100)	2 (100)	0	0
	IMP-22 + KPC-3 (2)	0	16	16	2 (100)	2 (100)	2 (100)	0	0
Eclo cx (22)	IMP-22 (7)	0	4	16	7 (100)	7 (100)	7 (100)	2 (28.6)	2 (28.6)
	IMP-13 (6)	3 (50)	4	16	6 (100)	6 (100)	5 (83.3)	3 (50)	1 (16.7)
	IMP-8 (5)	4 (80)	1	4	5 (100)	5 (100)	5 (100)	0	0
	IMP-22 + VIM-1 (2)	0	8	16	2 (100)	2 (100)	2 (100)	2 (100)	2 (100)
	IMP-15 (2)	0	4	4	2 (100)	2 (100)	1 (50)	0	0
Koxy (2)	IMP-28 (2)	0	4	4	2 (100)	2 (100)	2 (100)	2 (100)	2 (100)
Smar (1)	IMP-8	0	4	4	1 (100)	1 (100)	1 (100)	0	0
Eco (1)	IMP-13	1 (100)	2	2	1 (100)	1 (100)	1 (100)	0	0
Total		23 (46)	4	16	50 (100)	50 (100)	48 (96)	12 (24%)	26 (52%)

S, susceptible; CBPs, Carbapenemases; MHT, Hodge modified test; mCIM, modified carbapenemase inhibition method; DAI, dipicolinic acid inhibition. Kpn, *Klebsiella pneumoniae*; Eclo cx, *Enterobacter cloacae complex*; Koxy, *Klebsiella oxytoca*; Smar, *Serratia marcescens*; Eco, *Escherichia coli*.

^a^ Criteria as published by EUCAST (2022).

**Table 3:** Antibiotic susceptibility of 50 IMP-producing Enterobacterales as determined by the microdilution method according to EUCAST clinical breakpoints.

Antibiotics	S (%)	R (%)	MIC_50_^a^	MIC_90_^a^	Range^a^
Colistin	37 (74)	13 (26)	1	>8	0.5 to 8
Ceftazidime/Avibactam	0	50 (100)	>16	>16	>16 to 16
Amikacin	37 (74)	13 (26)	≤4	32	≤4 to 32
Imipenem	22 (44)	8 (16)	4	8	1 to 16
Meropenem	23 (46)	6 (12)	4	8	1 to 16
Gentamicin	4 (8)	46 (92)	>8	>8	2 to 8
Tigecycline^*^	-	-	4	>4	0.5 to 4
Trimethoprim/Sulfamethoxazole	16 (32)	34 (68)	>8	>8	≤1 to 8
Tobramycin	0	50 (100)	>8	>8	4 to 8
Aztreonam	20 (40)	26 (52)	2	>32	≤0.5 to 32
Ciprofloxacin	1 (2)	49 (98)	>2	>2	0.25 to 2
Ertapenem	0	50 (100)	>2	>2	1 to 2

S, susceptible, standard dosing regimen; R, resistant; MIC, minimum inhibitory concentrations.

a Expressed in mg/L.

* EUCAST has not breakpoints for tigecycline.

No IMP-Ent isolates were susceptible to ertapenem, more of them (62%) had MICs >2 mg/l. IMP-producing *K. pneumoniae* isolates showed high meropenem susceptibility rates, 75% (15 of the 20 isolates that produced only IMP carbapenemase) compared to other metallo-β-lactamases producers, such as NDM and VIM producers (Lutgring et al., 2020; Vázquez-Ucha et al., 2021). However, all isolates had MICs for meropenem within screening cut-off values recommended by EUCAST to suspect CPE (European Committee on Antimicrobial Susceptibility Testing [EUCAST], 2017), a key fact to consider for avoiding under-detection of IMP-producing *K. pneumoniae*.

According to carbapenemase type, four of the 20 (20%) isolates that produced only IMP-8 (14 *K. pneumoniae*, 4 *E. roggenkampii*, one *E. hormaechei*, one *S. marcescens*) were non-susceptible to meropenem; while 10 of the 13 (76.9%) isolates that produced only IMP-22 (7 *E. roggenkampii* and 6 *K. pneumoniae*) were non-susceptible to this antibiotic.

Table 3 shows antibiotic susceptibility of the 50 IMP-producing Enterobacterales. Regarding non-carbapenem antibiotics, the most active antibiotics tested were colistin (74% of susceptibility), amikacin (74%), aztreonam (40%), and trimethoprim/sulfamethoxazole (32%). Eleven of the 13 colistin-resistant isolates were *E. roggenkampii.* In a recent study with Enterobacterales from companion animals in Japan (Sato et al., 2022), many of them belonging to lineages with human clinical isolates, resistance to colistin was higher in Enterobacter spp. (20.3%) than in *K. pneumoniae* (1.2%) or *E. coli* (0%).

We performed different phenotypic tests for carbapenemase characterization and found all isolates were positive by the MHT and β-CARBA methods, regardless of species (Table 2). The mCIM was positive in 48 (96%) isolates, with a mean inhibition zone of 7.3 mm (range: 6–18 mm); two *E. hormaechei* isolates (IMP-13 and IMP-15) displaying intermediate results (16–18 mm) (Table 2). However, dipicolinic acid inhibition results yielded poor with only 12 (24%) positive isolates: four *E. roggenkampii*, three *E. hormaechei,* three *K. pneumoniae* and two *K. oxytoca*. The mean difference between inhibition zones observed with meropenem tablets (10 μg) and those obtained with meropenem plus dipicolinic acid tablets was 2.1 mm (range: 0–7 mm). This poor inhibition by dipicolinic acid could result from interference by chromosomal AmpC in *E. cloacae* complex isolates and, mainly, due to the slight hydrolytic activity of IMP carbapenemases against meropenem in *K. pneumoniae* (Doyle et al., 2012). However, the inhibition method using ertapenem plus EDTA showed that in 26 isolates (52%; 19 *K. pneumoniae*, 4 *E. roggenkampii*, 2 *K. oxytoca,* and one *E. hormaechei*), the activity of ertapenem was recovering with EDTA (increase of the inhibition zone ≥5 mm). Although not recommended by EUCAST guidelines, the use of ertapenem plus EDTA seems to be more sensitive for detection of IMP-Ent, mainly in *K. pneumoniae* isolates (19/24; 79.2%), than meropenem plus dipicolinic.

### Resistance, virulence and capsular polysaccharide *cps* loci by WGS

By WGS, we determined the profile of genes conferring resistance to antimicrobial agents (resistome) (Supplementary Table S1). An average of 11.2 antimicrobial resistance genes were detected in IMP-Ent isolates (range: 4–19), with small differences observed between *E. cloacae* complex (10.3; range: 7–18) and *K. pneumoniae* (12.8; range: 7–19) (Supplementary Table S1).

Acquired ESBLs or AmpC genes were detected in 15 (37.5%) IMP-Ent; 14 has ESBLs genes (12 *bla*_CTX-M-15,_ 1 *bla*_SHV-134_ and 1 *bla*_GES-7_) and one AmpC acquired genes (*bla*_FOX-5_) (Table 1). Chromosomal AmpC cephalosporinases genes were detected in *E. hormaechei* and *E. asburiae* (*bla*_ACT_), and in *E. roggenkampii* (*bla*_MIR_) (Supplementary Table S1). The prevalence of ESBL in IMP-producing strains (28%) was much lower than the 61.3% detected in a recent Spanish study with isolates producing any type of carbapenemase (Cañada-García et al., 2022); although, as in it, CTX-M-15 predominated. The different prevalence of ESBL and carbapenemase co-production according to Enterobacterales species (Arana et al., 2017; Cañada-García et al., 2022), usually higher in *K. pneumoniae,* was also detected in IMP-producing isolates, with only 11.5% of isolates co-producing IMP and ESBL in other species than *K. pneumoniae.*

Six *E. hormaechei* and one *E. asburiae* carried *mcr-9*.1 gene, but they were susceptible to colistin, as previously described (Macesic et al., 2021). No *mcr* genes were detected in colistin resistant isolates (11 *E. roggenkampii* and two *K. pneumoniae*). Cluster-dependent colistin resistance has been described in *Enterobacter cloacae* complex, mainly due to the expression of *pho*P/*pho*Q two-component regulatory system (Guérin et al., 2016).

All 14 IMP-producing *K. pneumoniae* isolates non-susceptible to aztreonam had additional carbapenemase or ESBL/AmpC acquired genes (Table 1). The high prevalence of aztreonam non-susceptibility in *E. cloacae* complex could be consistent with overproduction of chromosomal AmpC in these species.

IMP-producing *K. pneumoniae* isolates belonged to seven capsular polysaccharide *cps* loci (Supplementary Table S1), mainly KL151 (7; 29.2%), KL53 (5; 20.8%) and KL24 (4; 16.7%). High correlations between K-loci and STs have been previously described (Cañada-García et al., 2022); in this study all KL151 isolates were ST405 and all KL53 were ST464. The two *K. pneumoniae* isolates causing bacteremia belonged to unrelated KL24 and KL30 *cps* loci.

A total of eight isolates of *K. pneumoniae* presented yersiniabactin-encoding locus (*ybt*), six of them belonged to ST405, and the other two to ST788 and ST3293, respectively. In one ST405 isolate, yersiniabactin, aerobactin (*iuc*5 linage) and salmochelin (*iro*5 linage) were detected. *Ybt* has been previously related to virulence, particularly in pulmonary infection (Lawlor et al., 2007), but this association could not be proved in this study since 6 of the 8 isolates that carried it were implicated in colonizations, and only two were involved in urine infections.

### Phylogenetic analysis of IMP-producing *Enterobacter cloacae* complex and *Klebsiella pneumoniae*

CP-Kpn isolates were grouped into 7 STs (Table 1), being the most prevalent ST405 (*n* = 7; 29.2%), ST15 (*n* = 6; 25%) and ST464 (*n* = 5; 20.8%) which account for 75% of all IMP-producing *K. pneumoniae* isolates. Full correlation between STs ST405 and ST464 with K-locus KL151 and KL53, respectively, were observed. However, ST15 isolates belonged to two different K-locus (four KL24 and two KL112).

**Figure 1:**
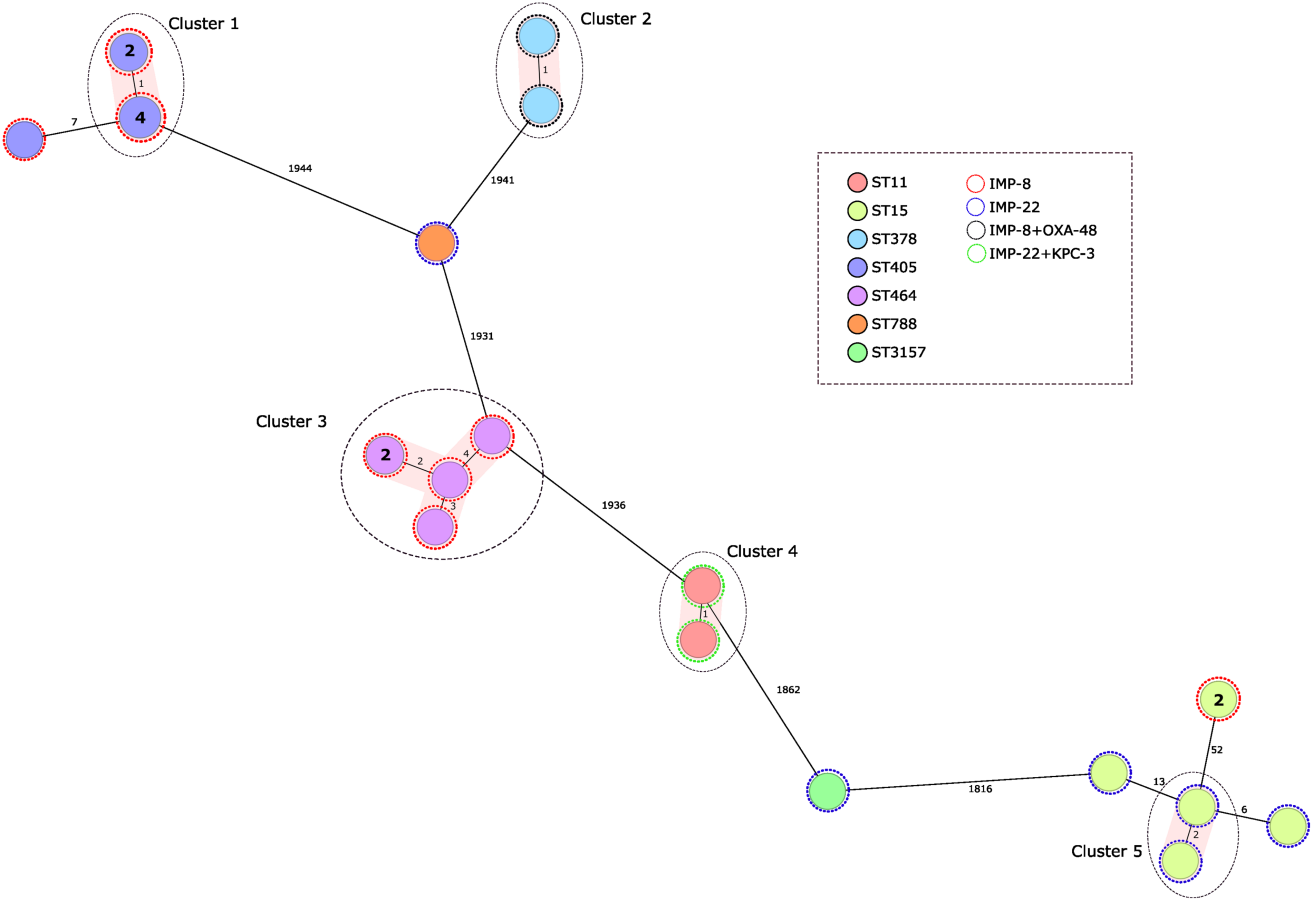
Population structure of IMP-producing *Klebsiella pneumoniae*: minimum-spanning tree. Distances shown are based on cgMLST of 2.358 genes using the parameter ‘pairwise ignoring missing values’. Fill colors in each circle indicate the MLST type and the color of the dashed line in circles indicates IMP type. Grey ovals represent clusters, applying a threshold of both 5 SNPs and 5 alleles.

Genome assemblies of the 24 IMP-producing *K. pneumoniae* isolates included in this study were analyzed using a gene-by-gene approach and the allelic distance from cgMLST was visualized in a minimum spanning tree (Figure 1). Five clusters were detected considering a genetic diversity ≤5 alleles (Figure 1). In only two of those clusters there have been grouped more than three isolates: Cluster 1 consisted of six ST405/IMP-8-produccing isolates submitted from a hospital located in the south of the country (four isolates were identical with the 2.538 alleles cgMLST scheme, and two presented one allele difference), these six isolates differed by an average of 2 SNPs (range 0–3); and Cluster 3 consisted in five ST464/IMP-8-produccing isolates submitted from the same hospital (average allelic distance: 4 alleles), these five isolates differed by an average of five SNPs (range 0–10). The average difference between Cluster 1 and Cluster 3 isolates was 20.489 SNPs and 1.961 alleles.

*K. pneumoniae* ST15 represents the only ST detected in two different provinces and encoding different *bla*_IMP_ genes (*bla*_IMP-8_, *bla*_IMP-22_). Notably, we did not detect ST14 or ST37, the types found to be most prevalent in an international study analyzing 22 IMP-producing *K. pneumoniae* isolates, all originating from Asia-Pacific countries (Matsumura et al., 2017). Rather, ST405 and ST15 predominated in our collection. Both have frequently been described in Spain in recent years, although they were associated with other carbapenemases, including OXA-48, for both ST405 and ST15 (Oteo et al., 2015), and VIM-1, for ST15 (Sánchez-Romero et al., 2012). However, other carbapenemases types continue to be prevalent in these STs (Oteo et al., 2015; Cañada-García et al., 2022); the appearance of IMP in different STs according to geographical location could be more related to sporadic transmission by mobile genetic elements between the predominant clones than to a true switch of the ST/carbapenemase type association.

Among IMP-producing *E. cloacae* complex isolates, 5 different STs were identified; all 13 *E. roggenkampii* belonged to ST96, *E. hormaechei* isolates belonged to the ST182 (*n* = 5), ST68 (*n* = 2) and ST90 (*n* = 1), and the *E. asburiae* isolate belonged to the ST484 (Table 1).

**Figure 2:**
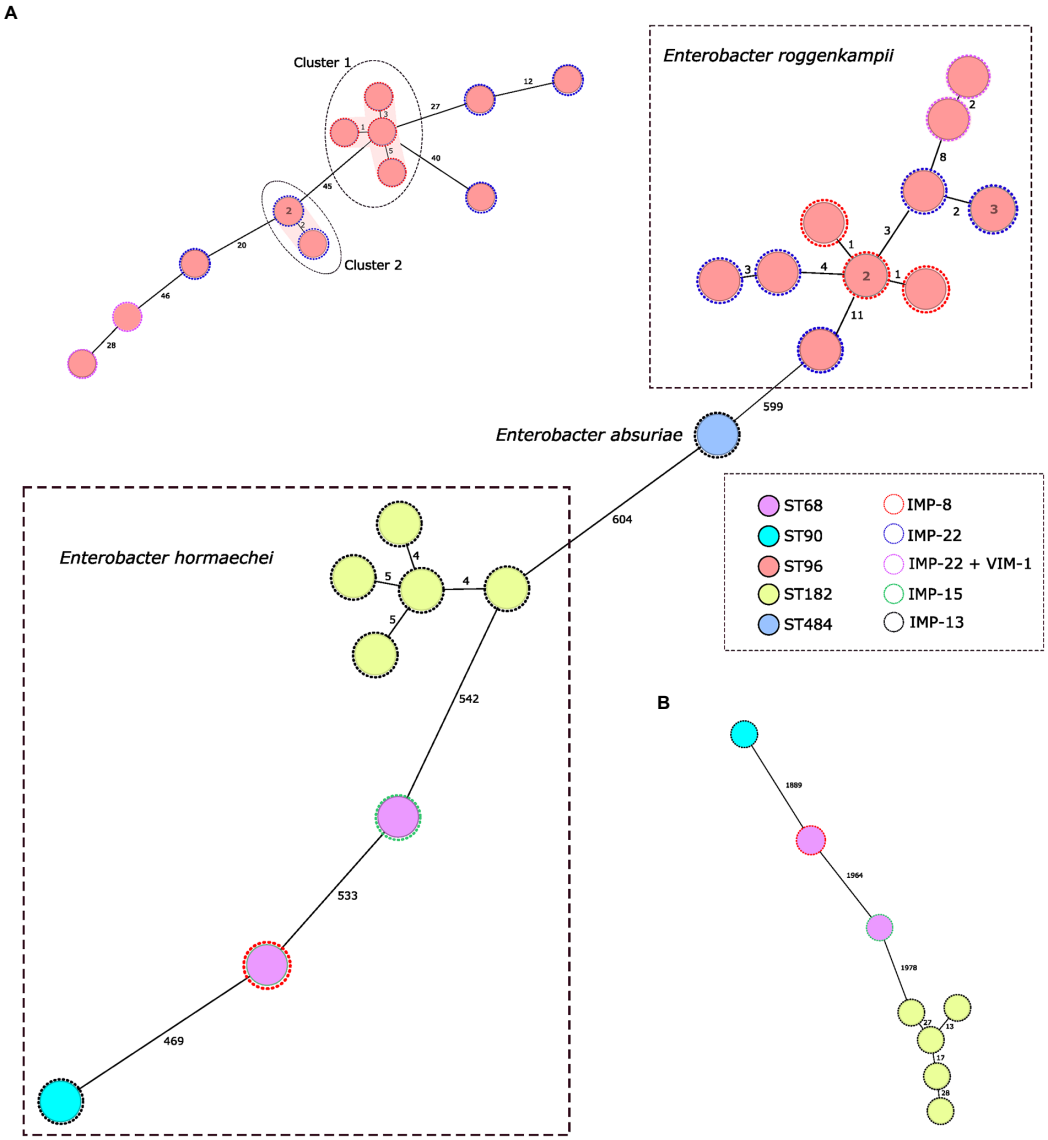
Population structure of IMP-producing *Enterobacter cloacae* complex: minimum-spanning tree. Distances shown are based on cgMLST of 631 genes using the parameter ‘pairwise ignoring missing values’. Fill colors in each circle indicate the MLST type and the color of the dashed line in circles indicates IMP type. **(A,B)** represent a minimum-spanning tree for each *Enterobacter* specie based on specific cgMLST of 2.123 and 2.466 genes, respectively, for *Enterobacter hormaechei* and *Enterobacter roggenkampii*. Grey ovals represent clusters, applying a threshold of both 5 SNPs and 5 alleles.

Genome assemblies of the 22 IMP producing *E. cloacae* complex isolates included in this study were analyzed using a gene-by-gene approach and the allelic distance from cgMLST obtained with the *E. cloacae* complex, *E. hormaechei* and *E. roggenkampii* schemes were visualized in a minimum spanning tree (Figure 2). Only two clusters were detected in *E. roggenkampii* considering a genetic diversity ≤ 5 alleles in the scheme specific of this specie (Figure 2A). Cluster 1 consisted in four ST96/IMP-8-producing isolates from a hospital in the north of the country (average allelic distance: 3 alleles) and Cluster 2 consisted in three isolates, two were identical and the other presented two allelic differences.

## Conclusion

We found that IMP-Ent isolates remain infrequent in Spain, although they have recently been circulating in hospitals from different geographic regions, leading to nosocomial outbreaks. In this study, IMP-8-producing *K. pneumoniae* and IMP-22-producing *E. roggenkampii* constitute the most frequent IMP-producing Enterobacterales in Spain. Some specific tests for the diagnosis of MBLs, such as inhibition with EDTA or dipicolinic acid, presented false negative results in some IMP-producing strains. Consequently, this remains a subject of microbiological and epidemiological interest and warrants active surveillance.

## The Spanish IMP study group

Other members of the Spanish IMP Study Group include, Esteban Aznar (Laboratorio BrSalud, San Sebastian de los Reyes, Madrid); José Leiva (Clínica Universitaria de Navarra); Begoña Palop (Hospital Regional de Málaga); José Luis Barrios (Hospital Universitario Cruces, Barakaldo, Vizcaya); Pilar Zamarrón (Hospital Virgen de la Salud, Toledo); Fernando Artiles (Hospital Universitario de Gran Canaria Dr. Negrín, Las Palmas de Gran Canaria); Luis López-Urrutia (Hospital Universitario Río Hortega, Valladolid); Beatriz Orden (Hospital Universitario Puerta de Hierro, Madrid); Susana Hernando (Hospital General de Segovia); and Rafael Carranza (Hospital General La Mancha Centro).

## Data availability statement

The data presented in the study are deposited in the European Nucleotide Archive (ENA) repository (https://www.ebi.ac.uk/ena), accession number PRJEB54568.

## Ethics statement

Ethical review and approval was not required for the study on human participants in accordance with the local legislation and institutional requirements. Written informed consent for participation was not required for this study in accordance with the national legislation and the institutional requirements.

## Author contributions

MP-V and JO-I conceived, designed and coordinated the study. JC-G, NG, ERA, VB, NL, AN, TC, NM-R, SG-C, JC, EC, BA, and the Spanish IMP Study Group collected isolates and performed the experiments. JC-G, NG, BA, MP-V, and JO-I wrote the manuscript. All authors have read, edited and approved the final manuscript.

## Funding

This research was supported by grants from the Instituto de Salud Carlos III (numbers PI18CIII/00030 and PI21CIII/00039).

This research was also supported by CIBER-Consorcio Centro de Investigación Biomédica en Red (CB21/13/00095 and CB21/13/000968), Instituto de Salud Carlos III, Ministerio de Ciencia e Innovación and Unión Europea – NextGenerationEU.

This work was supported by Plan Nacional de I + D + i 2013–2016 and Instituto de Salud Carlos III, Subdirección General de Redes y Centros de Investigación Cooperativa, Ministerio de Economía, Industria y Competitividad, Spanish Network for Research in Infectious Diseases (REIPI RD16CIII/0004/0002 and REIPI RD16/0016/0007) and co-financed by the European Development Regional Fund (EDRF), “A way to achieve Europe,” Operative program Intelligent Growth, 2014–2020.

## Conflict of interest

The authors declare that the research was conducted in the absence of any commercial or financial relationships that could be construed as a potential conflict of interest.

## Publisher’s note

All claims expressed in this article are solely those of the authors and do not necessarily represent those of their affiliated organizations, or those of the publisher, the editors and the reviewers. Any product that may be evaluated in this article, or claim that may be made by its manufacturer, is not guaranteed or endorsed by the publisher.

## Footnotes

^1^ http://quast.bioinf.spbau.ru/

^2^ https://www.ncbi.nlm.nih.gov/refseq/about/prokaryotes/

^3^ https://github.com/katholt/Kleborate

^4^ https://bigsdb.pasteur.fr/klebsiella/

^5^ https://cge.cbs.dtu.dk

^6^ https://github.com/katholt/Kleborate
