# Corrigendum: Phenotypic and molecular characterization of IMP-producing Enterobacterales in Spain: predominance of IMP-8 in *Klebsiella pneumoniae* and IMP-22 in *Enterobacter roggenkampii*

**DOI:** 10.3389/fmicb.2023.1331683

**Published:** 2023-11-24

**Authors:** Javier E. Cañada-García, Natalin Grippo, Eva Ramírez de Arellano, Verónica Bautista, Noelia Lara, Ana María Navarro, Teresa Cabezas, Nora Mariela Martínez-Ramírez, Silvia García-Cobos, Jorge Calvo, Emilia Cercenado, Belén Aracil, María Pérez-Vázquez, Jesús Oteo-Iglesias, The Spanish IMP Study Group

**Affiliations:** ^1^Laboratorio de Referencia e Investigación en Resistencia a Antibióticos e Infecciones Relacionadas con la Asistencia Sanitaria, Centro Nacional de Microbiología, Instituto de Salud Carlos III, Madrid, Spain; ^2^Escuela Internacional de Doctorado, Ciencias Biomédicas y Salud Pública - IMIENS (UNED), Madrid, Spain; ^3^Centro de Educación Médica e Investigaciones Clínicas “Norberto Quirno”, Buenos Aires, Argentina; ^4^Servicio de Microbiología, Hospital de Poniente, Almería, Spain; ^5^Servicio de Microbiología, Hospital Universitario de Guadalajara, Guadalajara, Spain; ^6^CIBER de Enfermedades Infecciosas (CIBERINFEC), Spanish Network for Research in Infectious Diseases (REIPI), Instituto de Salud Carlos III, Madrid, Spain; ^7^Servicio de Microbiología, Hospital Universitario Marqués de Valdecilla, Santander, Spain; ^8^Servicio de Microbiología, Hospital Universitario Gregorio Marañón, Madrid, Spain; ^9^CIBER de Enfermedades Respiratorias (CIBERES), Instituto de Salud Carlos III, Madrid, Spain

**Keywords:** antimicrobial resistant bacteria, carbapenem resistance, whole genome sequencing, surveillance, IMP carbapenemase, Enterobacterales

In the published article, there was an error regarding the affiliation for **Javier E. Cañada-Garc**í**a**. In addition to affiliation 1, a second affiliation should be included as follows:

^2^Escuela Internacional de Doctorado, Ciencias Biomédicas y Salud Pública - IMIENS (UNED), Madrid, Spain

The authors apologize for this error and state that this does not change the scientific conclusions of the article in any way. The original article has been updated.

